# A deep learning-based clinical-radiomics model predicting the treatment response of immune checkpoint inhibitors (ICIs)-based conversion therapy in potentially convertible hepatocelluar carcinoma patients: a tumor marker prognostic study

**DOI:** 10.1097/JS9.0000000000002322

**Published:** 2025-03-14

**Authors:** Zijian Lin, Weidong Wang, Yongcong Yan, Zifeng Ma, Zhiyu Xiao, Kai Mao

**Affiliations:** a Department of Hepatobiliary Surgery, Sun Yat-Sen Memorial Hospital, Guangzhou, Guangdong, China; b Guangdong Provincial Key Laboratory of Malignant Tumor Epigenetics and Gene Regulation, Sun Yat-sen Memorial Hospital, Sun Yat-Sen University, Guangzhou, Guangdong, China; c Department of Interventional Radiography, Sun Yat-Sen Memorial Hospital, Guangzhou, Guangdong, China; d Shanghai Public Health Clinical Center, Shanghai, China

**Keywords:** conversion therapy, deep learning, hepatocellular carcinoma, immune checkpoint inhibitor, immunotherapy, radiomics

## Abstract

**Background:**

The majority of patients with hepatocellular carcinoma (HCC) miss the opportunity of radical resection, making immune check-point inhibitors (ICIs)-based conversion therapy a primary option. However, challenges persist in predicting response and identifying the optimal patient subset. The objective is to develop a CT-based clinical-radiomics model to predict durable clinical benefit (DCB) of ICIs-based treatment in potentially convertible HCC patients.

**Methods:**

The radiomics features were extracted by pyradiomics in training set, and machine learning models was generated based on the selected radiomics features. Deep learning models were created using two different protocols. Integrated models were constructed by incorporating radiomics scores, deep learning scores, and clinical variables selected through multivariate analysis. Furthermore, we analyzed the relationship between integrated model scores and clinical outcomes related to conversion therapy in the entire cohort. Finally, radiogenomic analysis was conducted on bulk RNA and DNA sequencing data.

**Results:**

The top-performing integrated model demonstrated excellent predictive accuracy with an area under the curve (AUC) of 0.96 (95% CI: 0.94–0.99) in the training set and 0.88 (95% CI: 0.77–0.99) in the test set, effectively stratifying survival risk across the entire cohort and revealing significant disparity in overall survival (OS), as evidenced by Kaplan–Meier survival curves (*P* < 0.0001). Moreover, integrated model scores exhibited associations with sequential resection among patients who achieved DCB and pathological complete response (pCR) among those who underwent sequential resection procedures. Notably, higher radiomics model was correlated with MHC I expression, angiogenesis-related processes, CD8 T cell-related gene sets, as well as a higher frequency of TP53 mutations along with increased levels of mutation burden and neoantigen.

**Conclusion:**

The deep learning-based clinical-radiomics model exhibited satisfactory predictive capability in forecasting the DCB derived from ICIs-based conversion therapy in potentially convertible HCC, and was associated with a diverse range of immune-related mechanisms.

**HIGHLIGHTS**

- The first application of deep learning-based radiomics study in predicting the response of immune checkpoint inhibitors (ICIs)-based conversion therapy in potentially convertible HCC.
- Early decline of serum markers and dilated vasculature in the arterial phase as independent predictors of ICIs-based treatment response.
- The radiogenetic analysis establishes a correlation between model scores and immune-related biological mechanism.

## Introduction

Hepatocellular carcinoma (HCC) ranks as the third leading cause of mortality in oncology, with a relative 5-year survival rate of approximately 18%. Findings from the BRIDGE study indicate that over 55% of HCC cases in China fail to undergo radical surgical resection due to advanced stages at the time of initial diagnosis^[^1^]^. Advanced HCC with a high risk of recurrence, such as large tumor size^[^2,3^]^, multiple tumors^[^4^]^, and vascular invasion^[^5,6^]^, still have a poor prognosis after resection. These patients also experience a higher incidence of complications and postoperative mortality^[^7,8^]^. Nevertheless, some studies have suggested potential benefits of liver resection for these patients^[^9-11^]^. Patients with unresectable HCC are typically recommended to consider alternative treatment modalities according to established clinical guidelines, including transcatheter arterial chemoembolization (TACE), hepatic artery infusion chemotherapy (HAIC), and systemic pharmacotherapy^[^12,13^]^.

In recent years, significant progress has been made in nonsurgical therapies for advanced and unresectable HCC. Particularly, the combination of immune checkpoint inhibitors (ICIs) with tyrosine kinase inhibitors, antiangiogenic agents, and local therapy, such as TACE and HAIC, has demonstrated an enhanced objective response rate compared to previous modalities^[^14-16^]^. These findings suggest that conversion therapy, defined as utilizing nonsurgical treatments to downstage or convert unresectable (uHCC) into resectable HCC, represents a viable option for patients^[^17^].^

Nevertheless, the high heterogeneity of HCC may lead to limited response to ICIs-based treatment, presenting a significant challenge for conversion therapy in this context^[^18^]^. Inadequate response to conversion therapy can result in the progression of technically resectable HCC, thereby missing opportunities for surgical intervention. Furthermore, aggressive treatment approaches may potentially impair liver function. Therefore, it is imperative to develop a predictive model for assessing the response to ICI treatment and indentifying patients who are more likely to benefit from conversion therapy.

Previous research, encompassing HCC and other cancer types, has indicated that specific biomarkers, such as tumor mutation burden (TMB), tumor infiltrating lymphocytes (TILs), and microsatellite instability/defective mismatch repair (MSI/dMMR), and PD-L1, may serve as potential predictors of response to immunotherapy^[^19^]^. However, the acquisition of these biomarkers still requires invasive biopsies, particularly in the context of HCC where biopsy is not a standard procedure for diagnosis, further complicating their retrieval^[^13^]^. Several studies have utilized hematology, demography, or semantic features in radiology to develop predictive models for the treatment response to ICIs^[^20^]^. The advancement in medical image analysis has facilitated the transformation of digitally encrypted medical images into a wide range of quantitative features, providing valuable insights into tumor pathology and biological characteristics^[^21^]^, and it has been widely applied in predicting pathological phenotype^[^22,23^]^, treatment responses^[^24^]^, and prognosis of HCC^[^25,26^]^. To date, there have been limited studies endeavoring to construct radiomics models for predicting the response to ICIs treatment, without incorporating advanced technologies such as deep learning methods^[^27,28^]^. To the best of our knowledge, there is a lack of research on utilizing radiomics for predicting the response to ICIs-based treatment in potentially convertible HCC.

In this study, we have developed an integrated predictive model for the response to ICIs-based treatment by incorportating clinical data, semantic features in CT images, CT radiomics, and deep learning techniques. Additionally, we investigated the underlying biological mechanism associated with the model.

## Methods

This retrospective study received approval from the ethics committees of our institution and was conducted in compliance with the Declaration of Helsinki. Due to the retrospective nature of the study, informed consents were exempted.

This study was directed by REporting recommendations for tumor MARKer prognostic studies (REMARK)^[^29^]^.

### Study design

Between January 2019 and April 2023, a total of 203 potentially convertible HCC patients underwent ICIs-based conversion therapy in our center and were enrolled in this study. Potentially convertible HCC patients were defined as those with preserved liver function, technically unresectable tumors or technically resectable but not recommended for radical resection according to guidance^[^13^]^, and without extrahepatic metastases. Additionally, the patient’s liver functional status should be sufficient to withstand the potential sequential hepatectomy. Patients were randomly divided into a training set and a test set in a ratio of 3:1 (153 in training set and 50 in test set). Furthermore, we collected baseline CT and sequencing data of 36 patients from The Cancer Genome Atlas Liver Hepatocellular Carcinoma (TCGA-LIHC) and The Cancer Imaging Archive (TCIA) databases to form the radiogenomics cohort utilized for immune-related bioinformatic analysis.

The detailed inclusion criteria for this study are shown in Supplementary Appendix S1. http://links.lww.com/JS9/E9.

### CT image examination

All patients underwent pretreatment Contrast-enhanced Computer Tomography (CECT), and the specific details can be found in Supplementary Table S1. http://links.lww.com/JS9/E8.

We have conducted a comprehensive review of previous studies and incorporated semantic features into our model construction, which are potentially associated with the response to ICIs-based treatment^[^30,31^]^. Two radiologists who analyzed the images and evaluated semantic features were blinded to clinical data, ensuring a meticulous and unbiased evaluation in accordance with rigorous academic standards. Disagreements were resolved through thoughtful discourse. A detailed definition of semantic features can be found in Supplementary Figure S1. http://links.lww.com/JS9/E7.

### ICIs-based treatment regimens

The treatment regimens in this study consisted of combinations of ICIs and antiangiogenic agents, ICIs and interventional therapy, as well as a combination of ICIs, antiangiogenic agents, and interventional therapy. These treatment strategies were developed by experienced senior doctors with a minimum of 10 years of clinical expertise. The specific drug regimen and the decision to combine interventional therapy are determined based on careful consideration of the patient’s medical condition and preferences. Detailed treatment procedures can be found in Supplementary Appendix S2, S3. http://links.lww.com/JS9/E9.

### Evaluation of treatment response

Patients underwent CECT examinations at intervals of 1–2 months following the initiation of treatment for evaluation purposes. The response to ICIs-based treatment was assessed during each CECT examinations. Patients were categorized into two groups based on their treatment response: durable clinical benefit (DCB) and non-DCB groups. The DCB group included patients who demonstrated an objective response (OR) lasting more than 6 months according to the Modified Response Evaluation Criteria in Solid Tumors (mRECIST), while the non-DCB group comprised patients who either showed no response or had an objective response lasting less than 6 months. The overall survival assessment was conducted by censoring all patients at the last follow-up date, which was 30 September 2023.

### Model constrution

The details are shown in Supplementary Appendix S5–S8. http://links.lww.com/JS9/E9. Key parameter settings for deep learning training are shown in Supplementary Table S2. http://links.lww.com/JS9/E8. The structure diagram of deep learning model are shown in Supplementary Figure S2–S3. http://links.lww.com/JS9/E7.

### Evaluation of α-fetoprotein (AFP) response

The measurement of AFP was conducted prior to treatment and at a follow-up period of 3 weeks to 1 month after treatment. Subsequently, the decrease in AFP levels during the second assessment was compared with that observed during the initial assessment. The optimal threshold for evaluating the extent of AFP decline was determined by maximizing Youden’s index using the training set. A decrease in AFP exceeding this optimal threshold indicated a favorable response (AFP response), while a decrease below this threshold suggested an inadequate response (non-AFP response). Furthermore, individuals with pretreatment AFP levels <7 ng/ml were categorized as having normal AFP (designated as “AFP <7 ng/ml”).

### Development and evaluation of models

**Figure 1.**
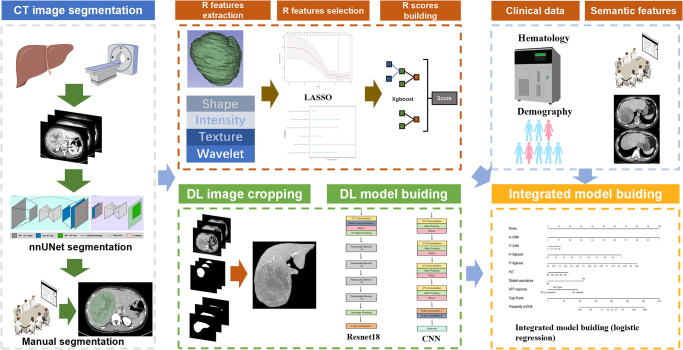
Workflow of CT segmentation, pretreatment, and model generation. LASSO, least absolute shrinkage and selection operator regression; CNN, convolutional neural network.

In order to identify the clinical information and semantic features utilized in constructing the nomogram, we conducted univariate and multivariable analyses comparing the DCB and non-DCB groups (Supplementary Appendix S4. http://links.lww.com/JS9/E9, Figure S4. http://links.lww.com/JS9/E7). Logistic regression was employed to develop clinical models incorporating various combinations of clinical variables, as well as an integrated model encompassing different combinations of clinical variables along with four radiomics scores. Besides, an integrated radiomics model solely based on the four radiomics scores was constructed for radiogenomics analysis. All models were assessed in the test set using receiver operating characteristic curve (ROC) analysis, calibration curve assessment (including Hosmer–Lemeshow test and Spiegelhalter *Z*-test), sensitivity, specificity, positive predictive value (PPV), negative predictive value (NPV). The protocols are shown in Fig 1.

### Radiogenomics analysis

The radiomics scores of samples in the radiogenomics cohort were calculated, and the median was used to stratify the sample into two groups: “DCB” and “Non-DCB”. Single-sample gene-set variation analysis (ssGSEA)^[^32^]^ was conducted to determine the gene-set enrichment score (GSE) for both the HALLMARK gene set^[^33^]^ and immune-related gene set^[^34^]^. A correlation analysis was performed between immune-related gene sets, HALLMARK gene sets, and radiomics scores, with only significant results (*P*-values < 0.05) being reported. Furthermore, an immune-related genomics analysis including tumor mutation burden (TMB) and neoantigen assessment was carried out between “DCB” and “Non-DCB” groups. TMB was defined as the number of mutations per megabase (Mut/Mb) of DNA using Maftools^[^35^]^, while neoantigens were predicted from single-nucleotide variants (SNV), following previous research^[^34^].^ Mann–Whitney U-test was employed to compare TMB and neoantigen levels between the “DCB” and “Non-DCB” groups. Additionally, we presented a list of top 10 genes exhibiting highest mutation rates within each group.

### Statistical analysis

For the analysis of clinical data and semantic features, continuous variables were assessed using *t*-tests and Mann–Whitney U-tests, while categorical variables were evaluated using Pearson χ^2^ tests or Fisher’s exact tests. Subsequently, multivariable logistic regression analyses were conducted to identify independent predictive variables. The model prediction performance was assessed using ROC, and the areas under the curve (AUC) were compared using the Delong test. Furthermore, the goodness-of-fit of the model was evaluated using Spiegelhalter *Z*-test and Hosmer-Lemeshow test. For survival analysis, patients were categorized into “DCB” and “Non-DCB” groups based on integrated models with an optimal threshold determined by maximizing Youden’s index in the training set. Kaplan–Meier (KM) method was employed to assess the overall survival (OS) between these two groups, with comparison performed using log-rank test. This study adopted a double-sided inspection approach with statistical significance defined as a *P*-value < 0.05. All statistics analysis were performed using R software (version 4.3.2; https://www.r-project.org/).

## Results

### Patient characteristics

**Figure 2.**
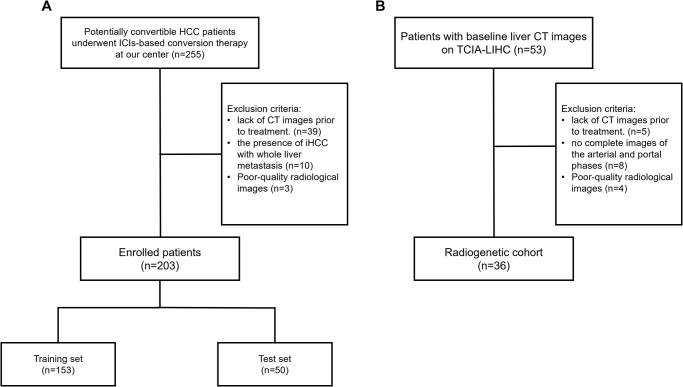
Flow chart of this study design.

Upon initial enrollment, a total of 255 patients who met the inclusion criteria were included in this study. Subsequently, 39 patients were excluded due to the absence of pretreatment CT images, while 10 patients were excluded because they had iHCC with whole-liver metastasis. Additionally, an additional three patients were excluded due to poor-quality radiological images (Fig. 2A). The median follow-up duration for both the training set and test set was found to be 21 months (95%CI, 18 to 22) and 21 months (95%CI, 18 to 26), respectively. Table 1 outlines the baseline characteristics of all enrolled patients. In the training set, out of a total of 153 patients, DCB was achieved in 93 cases; similarly, in the test set comprising of 50 patients, DCB was achieved in 35 cases. No statistical significance differences were observed between the baseline data of the training as well as test sets and different treatment regimes (Supplementary Table S3. http://links.lww.com/JS9/E8). On average, all enrolled patients demonstrated a maximum tumor diameter exceeding 90 mm with over 60% of them exhibiting vascular invasion. Moreover, nearly 90% of these individuals belonged to Child-Pugh grade A category indicating their locally advanced tumor burden along with preserved liver function rendering them unsuitable for surgical resection but capable enough to tolerate ICIs-based treatment.

**Table 1:** Baseline characteristics of potentially convertible HCC patients

	Train set	Valid set	*P -value*
	*N* = 153	*N* = 50	
Age, years			0.799
<60	99 (64.7%)	34 (68.0%)	
≥60	54 (35.3%)	16 (32.0%)	
Gender			0.204
Female	8 (5.23%)	0 (0.00%)	
Male	145 (94.8%)	50 (100%)	
BMI, means ± SD	22.7 ± 2.91	23.0 ± 2.94	0.545
Tumor size (mm), Mean ± SD	97.0 ± 38.0	96.2 ± 37.1	0.889
Numbers:			0.372
Multiple	113 (73.9%)	33 (66.0%)	
Single	40 (26.1%)	17 (34.0%)	
CNLC stage			0.592
1b–2a	38 (24.8%)	15 (30.0%)	
2b–3a	115 (75.2%)	35 (70.0%)	
ALBI:			0.579
1–2	117 (79.1%)	42 (84.0%)	
3	31 (20.9%)	8 (16.0%)	
Child-Pugh level			1
A	136 (88.9%)	45 (90.0%)	
B	17 (11.1%)	5 (10.0%)	
PS ECOG	0.27 ± 0.45	0.22 ± 0.42	0.434
Therapeutic regimens:			0.246
ICIs + Interventional treatment	23 (15.0%)	3 (6.00%)	
ICIs + antiangiogenic agents	15 (9.80%)	6 (12.0%)	
ICIs + antiangiogenic agents + Interventional treatment	115 (75.2%)	41 (82.0%)	
HBV:			0.739
No	9 (5.88%)	4 (8.00%)	
Yes	144 (94.1%)	46 (92.0%)	
Cirrhosis:			0.682
No	94 (61.4%)	33 (66.0%)	
Yes	59 (38.6%)	17 (34.0%)	
Vascular invasion:			0.858
No	57 (37.3%)	20 (40.0%)	
Yes	96 (62.7%)	30 (60.0%)	
Vp:			1
Vp1–Vp2	11 (12.8%)	3 (11.5%)	
Vp3–Vp4	75 (87.2%)	23 (88.5%)	
PLT (× 10^9^)	222 ± 110	206 ± 88.3	0.308
Creatinine (μmol/l)	80.1 ± 16.0	84.6 ± 15.1	0.075
TB (μmol/l)	21.8 ± 13.8	19.8 ± 11.9	0.324
DBIL (μmol/l)	7.77 ± 7.94	6.48 ± 6.01	0.231
INR	1.07 ± 0.10	1.25 ± 1.47	0.407
AFP (ng/ml):			0.977
≤ 400	68 (44.4%)	23 (46.0%)	
> 400	85 (55.6%)	27 (54.0%)	
AFP response:			0.41
AFP responder	95 (62.1%)	26 (52.0%)	
AFP<7 ng/ml	19 (12.4%)	9 (18.0%)	
Non-AFP responder	39 (25.5%)	15 (30.0%)	
Intratumoral necrosis			0.6
No	71 (46.4%)	26 (52.0%)	
Yes	82 (53.6%)	24 (48.0%)	
Peritumoral enhancement			0.36
No	124 (81.0%)	44 (88.0%)	
Yes	29 (19.0%)	6 (12.0%)	
Regular margin			0.297
No	70 (45.8%)	18 (36.0%)	
Yes	83 (54.2%)	32 (64.0%)	
Pseudocapsule			0.351
No	96 (62.7%)	27 (54.0%)	
Yes	57 (37.3%)	23 (46.0%)	
Dilated vasculature			0.566
No	61 (39.9%)	17 (34.0%)	
Yes	92 (60.1%)	33 (66.0%)	
Treatment response			
DCB	93 (60.8%)	35 (70.0%)	0.316
Non-DCB	60 (39.2%)	15 (30.0%)	
mRECIST			0.333
CR	20 (13.1%)	7 (14.0%)	
PR	73 (47.7%)	28 (56.0%)	
SD	10 (6.54%)	5 (10.0%)	
PD	50 (32.7%)	10 (20.0%)	

Continuous variables were reported as mean ± standard deviation (SD) and compared using either the *t*-test or Mann–Whitney U-test, while categorical variables were presented as frequencies with percentages and compared using the chi-squared test. Statistically significant correlations are indicated in bold text with a *P*-value < 0.05.

BMI, body mass index; Tumor size, the maximum diameter of the largest tumor; CNLC, China Liver Cancer Staging; ALBI, albumin–bilirubin; ICIs, Immune checkpoint inhibitors; PS, performance status; ECOG, Eastern Cooperative Oncology Group; HBV, viral hepatitis type B; Vp, The portal vein invasion classification system of the Liver Cancer Study Group of Japan; AFP, **α**-fetoprotein; INR, international normalized ratio; TB, total bilirubin; DBIL, direct bilirubin; PLT, platelet, DCB, durable clinical benefit; mRECIST, treatment response at 6 months after treatment according to Modified Response Evaluation Criteria in Solid Tumors (mRECIST). CR, complete response; PR, partial response; SD, stable disease; PD, progression disease.

### Univariate and multivariate analysis

The optimal threshold of AFP response was 65% and was set to be 60% for a simpler application (Supplementary Figure S4. http://links.lww.com/JS9/E7). Regarding baseline data, patients with CNLC stage Ib–IIa had a better response to ICIs-based treatment compared to those with IIb–IIIa (OR 0.4, 95% CI 0.16 to 0.89). Additionally, it was observed that patients with low platelet count exhibited an enhanced response to ICIs-based treatment (OR 1, 95% CI, 0.99 to 1.00). Semantic features in radiology revealed that patients who achieved DCB showed more frequent dilated vasculature in arterial phase (OR 2.5 95%CI, 1.28 to 4.95), as well as regular margins (OR 3.67 95% CI, 1.87 to 7.40), and less frequent peritumoral enhancement (OR 0.38 95% CI, 0.16 to 0.86). Importantly, patients with AFP response were more likely to achieve DCB. Interestingly, no statistically significant difference was observed between the DCB and Non-DCB groups for commonly used prognostic variables such as baseline AFP levels (OR 0.74 95% CI, 0.38 to 1.44), tumor size (OR 1 95% CI, 0.99 to 1), and number of tumors (OR 1.47 95% CI, 0.69 to 3.24).

The multivariate analysis demonstrated that AFP response (OR 2.35; 95% CI, 1.20 to 4.65, *P* = 0.013), platelet count (0.99; 95% CI, 0.99 to 1.0, *P* = 0.030), and dilated vasculature in arterial phase (OR, 2.69; 95% CI, 1.38 to 5.31, *P* = 0.003) independently predict DCB (Table 2).

**Table 2:** Univariate and multivariate analysis of clinical variables in training set

	Univariate analysis				Multivariate analysis		
Variables	OR	95% CI	*P-*value		OR	95% CI	*P-*value
Age, years							
<60							
≥60	1.89	0.94–3.91	0.075				
Gender							
Female							
Male	0.53	0.07–2.46	0.435				
BMI	0.98	0.88–1.10	0.766				
Tumor size (mm)	1	0.99–1.00	0.255				
Numbers							
Multiple							
Single	1.47	0.69–3.24	0.32				
CNLC stage							
1b–2a							
2b–3a	0.4	0.16–0.89	0.024		0.60	0.26–1.35	0.231
ALBI							
1–2							
3	1.02	0.45-2.37	0.959				
Child-Pugh level							
A							
B	0.54	0.19–1.51	0.236				
ECOG	0.54	0.26–1.11	0.095				
Therapeutic regimens							
ICIs + Interventional treatment							
ICIs + antiangiogenic agents	1.27	0.32–5.39	0.737				
ICIs + antiangiogenic agents + Interventional treatment	0.97	0.37–2.42	0.948				
HBV							
No							
Yes	1.26	0.29–5.16	0.743				
Cirrhosis							
No							
Yes	0.72	0.37–1.41	0.337				
Vp:							
Vp1–Vp2							
Vp3–Vp4	0.63	0.15–2.33	0.495				
Vascular invasion							
No							
Yes	0.53	0.26–1.05	0.069				
PLT (× 10^9^)	0.99	0.99–1.00	0.039		0.99	0.99-0.99	0.030
Creatinine (μmol/l)	0.98	0.96–1.00	0.06				
TB (μmol/l)	1.01	0.98–1.03	0.511				
DBIL (μmol/l)	0.98	0.94–1.02	0.357				
INR	6.41	0.21–195	0.286				
NLR	0.91	0.81-1.02	0.071				
AFP:							
≤400							
>400	0.74	0.38–1.44	0.381				
AFP response							
Non-AFP responder							
AFP <7 ng/ml	3.3	1.10–11.50	0.033		1.25	0.47–3.53	0.656
AFP responder	2.92	1.44–6.05	0.003		2.35	1.20–4.65	0.013
Intratumoral necrosis							
No							
Yes	0.91	0.47–1.75	0.783				
Peritumoral enhancement							
No							
Yes	0.38	0.16–0.86	0.021		0.57	0.25–1.31	0.190
Regular margin							
No							
Yes	3.67	1.87–7.40	<0.001		1.67	0.85–3.28	0.130
Enhancing capsule							
No							
Yes	1.89	0.95–3.88	0.069				
Dilated vasculature							
No							
Yes	2.5	1.28–4.95	0.007		2.69	1.38–5.31	0.003

BMI, body mass index; Tumor size, the maximum diameter of the largest tumor; CNLC, China Liver Cancer Staging; ALBI, albumin–bilirubin; ICIs, Immune checkpoint inhibitors; PS, performance status; ECOG, Eastern Cooperative Oncology Group; HBV, viral hepatitis type B; Vp, The portal vein invasion classification system of the Liver Cancer Study Group of Japan; AFP, **α**-fetoprotein; INR, international normalized ratio; TB, total bilirubin; DBIL, direct bilirubin; PLT, platelet, AFP response; The decrease in **α**-fetoprotein at one month after treatment greater than the optimal threshold obtained by ROC analysis.

### Radiomics and deep learning model construction

**Figure 3.**
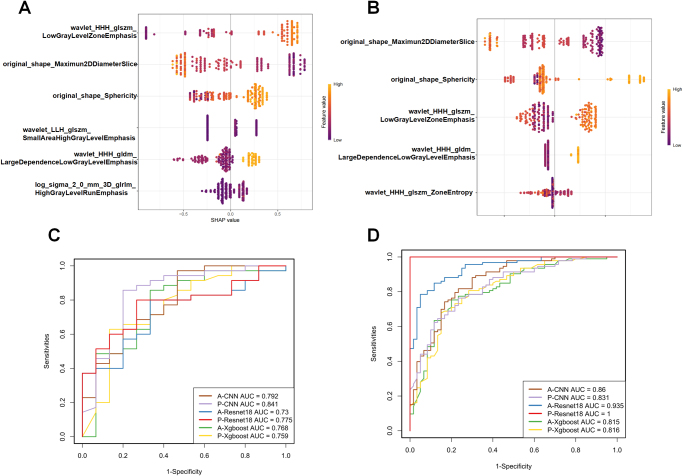
Radiomics features importance and performance of the Xgboost radiomics models for predicting DCB. (A) Bee swarm plot of radiomics features from Xgboost model in arterial phase. The X-axis represents the SHAP value of the variable, and the color of the dot represents the original value of the variable. (B) Bee swarm plot of radiomics features from Xgboost model in portal phase. (C–D) Performance of the radiomics and deep learning models with ROC analysis in the training set (C), and test set (D).

The radiomics features were extracted and selected separately from images in arterial phase and portal phase, following the aforementioned protocol. Xgboost was employed for generating the machine learning models and developing the radiomics score in both phases (defined as “A-Xgboost” and “P-Xgboost”). Figure 3A–B illustrates the selected features and their SHAP value. In the arterial phase model, wavelet_HHH-glszm_LowGrayLevelZoneEmphasis was positively correlated with DCB while original_shape_Maximum2DDiameterSlice was negatively corgrelated with DCB. The same was observed in the portal-phase model.

The described methods were applied to construct deep learning models in the arterial phase and portal phase. The CNN model demonstrated superior performance compared to the resnet18 models in the test set, thus it was selected for building the deep learning score referred to as “A-CNN” and “P-CNN” (Fig. 3C–D) (Supplementary Table S4. http://links.lww.com/JS9/E8).

An integrated radiomics model were generated by incorporating four radiomics scores through multivariate logistic regression analysis.

### Clinical models and integrated nomogram construction and evaluation

Clinical models were generated using different combinations of clinical independent predictors selected through multivariate analysis, while integrated models were constructed by incorporating both clinical independent predictors and four radiomics scores. Notably, the assessment of AFP response is only available one month after treatment initiation. Therefore, we developed clinical models and integrated models with or without AFP response. We computed the Variance Inflation Factor (VIF) of each variable in the Integrated model, and the results indicated that there was no significant multicollinearity in the model (Supplementary Table S12. http://links.lww.com/JS9/E8).

**Figure 4.**
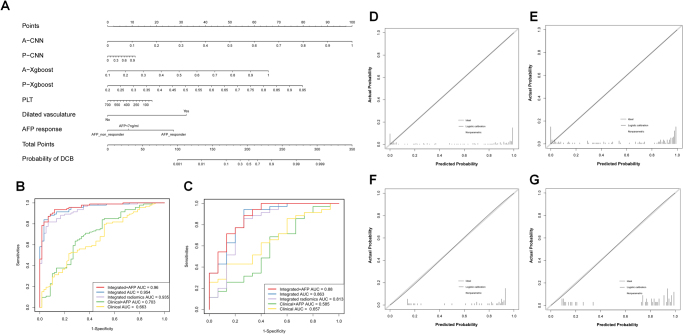
Construction and evaluation of the integrated and clinical models for predicting DCB. (A) Nomogram of the integrated model with AFP response, scaled by the regression coefficient of each variable, and displaying the variable contributions. (B–C) Performance of the integrated and clinical models models with ROC analysis in the training set (B), and test set (C). (D–G) Calibration curves of predicted DCB probability and actual probability in integrated model with AFP response in training set (D) and test set (E), integrated model without AFP response in training set (F) and test set (G).

The nomogram depicting the integrated model with AFP response is presented in Figure 4A. Both the integrated model and integrated radiomics model exhibited robust predictive capabilities for ICIs-based treatment. In training set, the integrated radiomics model achieved an AUC of 0.93 (95% CI: 0.9–0.97), while in the test set it achieved an AUC of 0.82 (95% CI: 0.66–0.97). On the other hand, the integrated model with AFP response attained an AUC of 0.96 (95% CI: 0.94–0.99) in the training set and an AUC of 0.88 (95%CI: 077 ~ 0.99) in the test set, whereas the integrated model without AFP response yielded an AUC of 0.96 (95% CI: 0.93 ~ 0.98) in training set and an AUC of 0.86 (95% CI: 0.75–0.98) in test set (Table 3, Fig 4B-C). Subgroup analysis revealed that our model exhibited satisfactory predictive performance across various regimens based on ICIs. Details are shown in Supplementary Figures S5–S6. http://links.lww.com/JS9/E7.

**Table 3:** Performance of different models

Dataset	AUC	Sensitivity(%)	Specificity(%)	PPV(%)	NPV(%)
Clinical model without AFP response					
Train set	0.66 (0.58, 0.75)	0.52 (0.41, 0.62)	0.77 (0.64, 0.87)	0.77 (0.65, 0.87)	0.51 (0.40, 0.61)
Test set	0.66 (0.5, 0.82)	0.43 (0.26, 0.61)	0.87 (0.60, 0.98)	0.88 (0.64, 0.99)	0.39 (0.23, 0.58)
Clinical model with AFP Response					
Train set	0.70 (0.61, 0.79)	0.65 (0.54, 0.74)	0.72 (0.59, 0.83)	0.78 (0.67, 0.87)	0.57 (0.45, 0.68)
Test set	0.58 (0.40, 0.77)	0.69 (0.51, 0.83)	0.53 (0.27, 0.79)	0.77 (0.59, 0.90)	0.42 (0.20, 0.67)
Radiomics model					
Train set	0.93 (0.9, 0.97)	0.82 (0.72, 0.89)	0.93 (0.84, 0.98)	0.95 (0.88, 0.99)	0.77 (0.65, 0.86)
Test set	0.82 (0.66, 0.97)	0.86 (0.70, 0.95)	0.73 (0.45, 0.92)	0.88 (0.73, 0.97)	0.69 (0.41, 0.89)
Integrated model with AFP response					
Train set	0.96 (0.94, 0.99)	0.92 (0.85, 0.97)	0.92 (0.82, 0.97)	0.95 (0.88, 0.98)	0.89 (0.78, 0.95)
Test set	0.88 (0.77, 0.99)	0.89 (0.73, 0.97)	0.73 (0.45, 0.92)	0.89 (0.73, 0.97)	0.73 (0.45, 0.92)
Integrated model model without AFP response					
Train set	0.96 (0.93, 0.98)	0.92 (0.85, 0.97)	0.92 (0.82, 0.97)	0.95 (0.88, 0.98)	0.89 (0.78, 0.95)
Test set	0.86 (0.75, 0.98)	0.94 (0.81, 0.99)	0.73 (0.45, 0.92)	0.89 (0.75, 0.97)	0.85 (0.55, 0.98)

Data in parentheses are 95% confidence interval (CI)

PPV, positive predictive value; NPV, negative predictive value.

The two integrated models exhibited significantly superior AUC values compared to the integrated radiomics model in both the training and test sets, except for the integrated model without AFP in the test set. Notably, including AFP response in the integrated model did not result in a statistically significant impact on predictive performance. However, the clinical model incorporating AFP response showed higher sensitivity, while the clinical model without AFP response exhibited higher specificity. Additionally, Delong tests confirmed that both the integrated radiomics model and integrated model outperformed the clinical model with statistically significant differences in AUCs. Detailed results of Delong test are provided in Supplementary Table S5. http://links.lww.com/JS9/E8.

The calibration curves of the two integrated models demonstrate robust agreement between predicted outcomes and observed data in both the training and validation cohorts (Fig. 4D-G). The Hosmer-Lemeshow test indicated no significant deviation from a perfect fit (*P* = 0.898 and 0.877 for the integrated model without AFP, *P* = 0.825 and 0.972 for the integrated model with AFP in the training and test sets, respectively). Additionally, the Spiegelhalter i-test yielded p-values of 0.913 and 0.892 for the integrated model without AFP, as well as *P*-values of 0.818 and 0.874 for the integrated model with AFP in both training and test sets, respectively.

In summary, integrated models exhibited the highest predictive capability, while the integrated radiomics model also demonstrated a satisfactory performance

### Models for further clinical application

**Figure 5.**
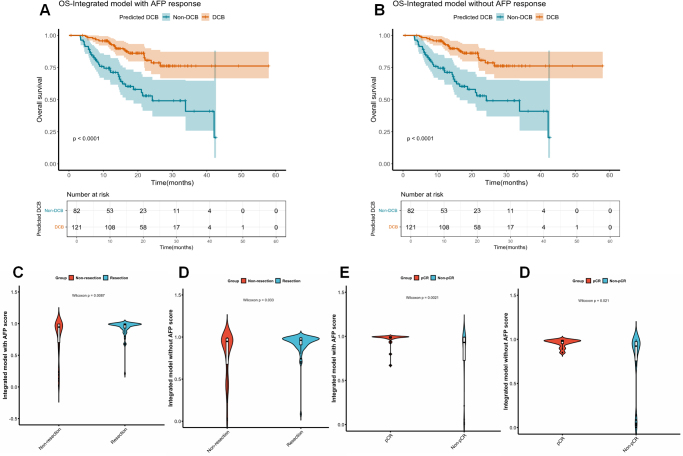
The further clinical application of the integrated models. (A–B) The Kaplan–Meier curves of overall survival according to the predicted DCB by the integrated model with AFP response (A) and without AFP response (B). (C–D) The relationship between the integrated model scores with AFP response (C) as well as without AFP response (D) and the sequential resection among patients achieving DCB in the entire cohort shown by violin plot. (E–F) The relationship between the integrated model scores with AFP response (E) as well as without AFP response (F) and the pCR among patients underwent sequential resection in the entire cohort shown by violin plot.

We conducted an analysis to evaluate the integrated model’s capacity in identifying overall survival (OS) benefits. In the entire cohort, the predicted non-DCB patients by the integrated model, with or without AFP response, exhibited a median OS of 24.2 months. However, the predicted DCB patients by the integrated model, with or without AFP response, did not reach a median survival outcome. The Kaplan–Meier survival curves revealed a statistically significant disparity in OS between identified DCB and identified Non-DCB patients using both integrated models (p<0.0001 for both two integrated model) (Fig. 5A-B).

The potential of model scores in guiding conversion therapy was assessed through a univariate analysis. Patients achieved successful conversion demonstrated higher scores across all three radiomics models (Fig. 5C–D, Supplementary Table S6. http://links.lww.com/JS9/E8). Additionally, for patients underwent sequential resection, univariate analysis revealed a correlation between pCR and two integrated model score as along with AFP response (Fig. 5E-F, Supplementary Table S7. http://links.lww.com/JS9/E8). Furthermore, The analysis between different mRECIST states demonstrated that our models have ability to differentiate between different mRECIST categories (Supplementary Table S8–S11. http://links.lww.com/JS9/E8). In addition, our model demonstrated promising outcomes in predicting the treatment response of patients under various treatment regimens (Supplementary Table S13-14. http://links.lww.com/JS9/E8). Overall, our models were able to provide guidance for the clinical application of conversion therapy.

### Radiogenomics analysis and deep learning model visualization

**Figure 6.**
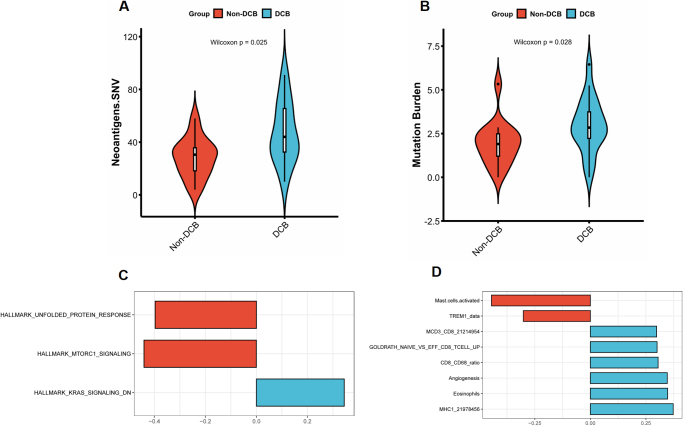
Radiogenetic analysis. (A–B)Violin plot shows the significantly significant higher level of neoantigens (A) and mutation burden (B) in DCB group predicted by integrated radiomics model. (C–D) Bar plots shows the significantly correlated gene sets with the integrated radiomics model score based on the gene set variation analysis scores of hallmark gene sets (C) and immune-related gene sets (D).

The immunogenetic analysis demonstrate a statistically significant higher level of neoantigens in the DCB group compared to the non-DCB group (Fig. 6A). The genome analysis revealed that the tumor mutation burden (TMB) was found to be statistically significantly higher in the DCB-group than in the Non-DCB group (Fig. 6B), while no statistically significant differences were identified in homologous recombination deficiency (HRD), intratumoral heterogeneity (ITH), copy number variation (CNV), and aneuploidy (Supplemental Figure S7. http://links.lww.com/JS9/E7). The GSEA showed associations between high radiomics model score and MHC I expression, angiogenesis, CD8_CD68 ratio as well as downregulation genes of KRAS signaling; whereas low radiomics model score was associated with mast cell activation, unfold protein response, mTOR signaling pathways (Fig. 6C-D). Moreover, we compared the mutation spectrum between the DCB and Non-DCB groups and presented top 20 mutation genes in Supplemental Figure S8. http://links.lww.com/JS9/E7. The DCB group exhibited a higher frequency of TP53 mutations, while there was no statistically significant disparity in CTNNB1 mutations between the two groups.

**Figure 7.**
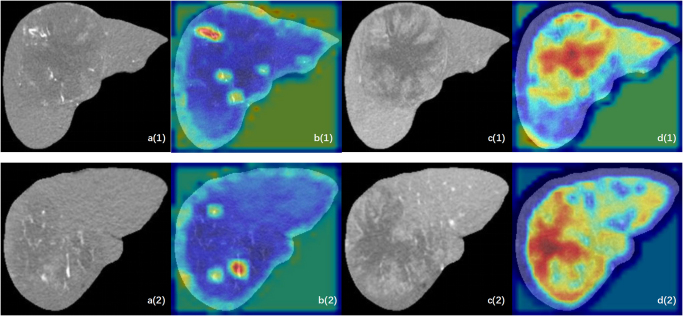
The GradCAM analysis of two patients. Warm colors contribute more significantly to the predictive classification. In contrast, blue and cold colors show a weaker correlation with the prediction. Patients (1) were predicted DCB and patients (2) were predicted Non-DCB. (A) The original CT image in arterial phase. (B) The GradCAM image in CNN model in arterial phase. (C) The original CT image in portal phase. (D) The GradCAM image in CNN model in portal phase.

The deep learning model in the arterial phase demonstrated a stronger emphasis on the dilated tumor vessels (Fig. 7a–b), while the model in the portal phase exhibited a greater concentration on the textural characteristics of the tumor (Fig. 7c-d). Interestingly, the no enhancement zone observed during the portal phase was deemed significant by this specific model (Fig. 7c-d). Overall, these findings highlight how the deep learning models focus on distinct information within CT images across different phases, thereby enhancing both diversity and comprehensiveness of information obtained by our integrated model.

## Discussion

Previous studies have used clinical information and hematological data to construct prediction models for treatment response and prognosis based on ICIs^[^20,36^]^. Furthermore, two radiomics studies exclusively employed CT radiomics features or deep learning features extracted from pretrained model^[^37^]^. Our study developed a comprehensive model incorporating machine learning, deep learning and clinical data to predict the response of ICIs-based conversion therapy combining in potentially convertible HCC patients. Both our integrated radiomics model and integrated model demonstrated excellent predictive ability, effectively reflecting pathological response and the feasibility of surgical resection. Furthermore, our integrated radiomics model successfully stratified patients into two distinct subgroups with divergent overall survival outcomes, indicating its potential for not only predicting treatment response but also assessing survival benefits. Importantly, through radiogenomics analysis, we identified immune-related biological mechanisms associated with our model’s performance. We are currently unaware of any radiomics research utilizing deep learning techniques to predict the efficacy of ICIs-based conversion therapy in potentially convertible HCC patients.

**Figure 8.**
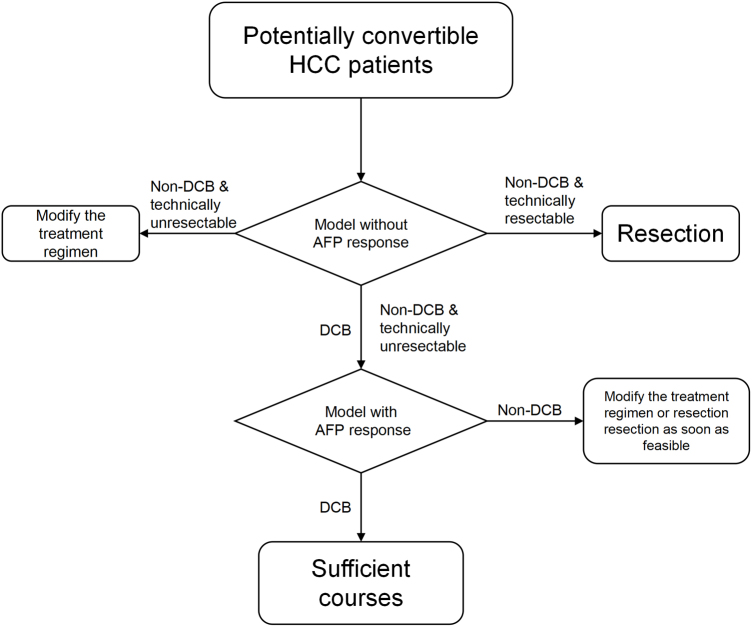
Streamlined clinical workflow of model.

The automated segmentation model we trained can assist doctors in rapid contouring. Once the contoured mask and the original CT files are obtained, feature extraction can be carried out using the pyradiomics software, and 2D images for deep learning can be cut through specific code. Subsequently, these images are fed into the model to obtain the Radiomics score and the deep learning score. Finally, clinicians collect clinical data and evaluate the semantic features of CT image, and then input these data into the integrated model and obtain predictive results. Regarding the clinical applicability, our integrated model demonstrated high predictive performance and can identify patients who were not sensitive to ICIs-based therapy and whose objective response did not last long enough. A 6-month period is commonly observed in clinical practice as the timeframe from the initiation of conversion therapy to the performance of radical surgical resection, typically not exceeding one year. Patients who are predicted by the model to achieve an objective response and maintain it for at least 6 months, thereby reaching a DCB, are sensitive to immune checkpoint inhibitors (ICIs)-based conversion therapy and are less likely to experience short-term tumor progression during treatment that would result in the loss of surgical opportunities. Clinicians can actively administer a sufficient course of ICIs-based conversion therapy to these patients, aiming to eliminate as many tumor cells as possible. Following a more thorough treatment response, surgical resection can be performed to reduce postoperative recurrence rates and achieve longer recurrence-free survival (RFS) and OS. For patients predicted by the model to be insensitive to ICIs-based therapy or unable to sustain an objective response for a sufficiently long period (6 months), clinicians can recommend alternative drug regimens or enrollment in clinical trials of new drugs to enhance the likelihood of achieving better treatment outcomes, thereby avoiding the adverse reactions associated with ICIs-based therapy. If ICIs-based conversion therapy is still employed, clinicians should closely monitor the patient’s tumor status. Once the tumor responds to treatment and meets the criteria for surgical resectability, radical resection should be performed as early as possible to prevent early tumor progression that could lead to the loss of surgical opportunities. For patients in this category who do not exhibit a significant treatment response, ICIs can be discontinued to mitigate potential adverse reactions (Fig. 8).

Deep learning method relying on preset formulas, demonstrating strong generalization capability. However, its optimal performance requires a substantial volume of training samples. Due to privacy regulations and challenges in data acquisition and labeling, the sample size of radiomics studies is limited. To maximize the utility of limited data, researchers have explored strategies such as transfer learning, and designing more efficient model architectures with low-parameter. These approaches aim to mitigate overfitting risk and enhance generalization performance. In our study with a small sample research (153 training samples), we designed a CNN model with fewer parameters. The superior performances of CNN compared to Resnet18 may be attributed to the need for converting grayscale CT images to RGB images required by pretrained Resnet18 models, which can result in loss of important details. Additionally, our model possesses an appropriate level of depth and complexity that helps mitigate overfitting caused by insufficient sample size. Moreover, we segmented the entire liver image from the slice containing the tumor’s maximum cross-sectional area is located and used it for training, which allows the model to extract information beyond the tumor itself, such as the surrounding area of the tumor, vascular invasion, tumor distribution, and the degree of liver cirrhosis. Overall, the application of deep learning has achieved promising results in our study.

Previous studies have demonstrated a correlation between the decline of AFP levels within one month after treatment and the response to immune checkpoint therapy^[^38,39^]^. In our study, we defined the optimal threshold as 60% based on the best Youden’s Index obtained from ROC analysis. For potentially convertible HCC patients, a more substantial reduction in AFP levels may be required to indicate a favorable treatment response. However, incorporating AFP response into our models did not significantly enhance their predictive capacity, possibly due to the robust predictive capability of the radiomics model and other clinical data. Several studies demonstrated that baseline AFP level were associated with therapeutic efficacy for HCC patients treated with ICI-based regimens^[^20^]^. However, we also reviewed the studies about locoregional interventions and their combination with immune immune checkpoint inhibitors and antiangiogenic agents/TKIs. These studies have a similar conclusion to our study that baseline AFP levels are not associated with the efficacy of treatment and prognosis^[^37,40,41^]^. The treatment response of ICIs-based therapy combining with locoregional interventions may not be affected by AFP baseline due to its its potent efficacy, but AFP response is still predictive of efficacy.

In terms of CT semantic features, dilated vasculature in arterial phase, which serves as an indicator related to blood vessels, was initially identified as a predictive factor for the response to ICIs-based treatment in this study. Dilated vasculature in arterial phase is considered a marker of TP53 mutation and vessel-encapsulating tumor cluster (VETC)^[^31^]^. VETC represents a recently defined characteristic vascular transformation mediated by angiogenesis and has been associated with a favorable treatment response to sorafenib^[^42^]^. However, recent studies have suggested that patients with VETC-positive HCC exhibit poor responses to HAIC and have worse prognoses^[^43,44^]^. The prognostic role of VETC in HCC remains controversial. HCC with a positive VETC pattern demonstrates higher expression levels of PD-L1 and fibroblast growth factor receptors, along with more efficient tumor metastasis. This suggests that HCC exhibiting VETC positivity may present a greater tumor burden while also displaying increased sensitivity to ICIs and antiangiogenic agents. Importantly, our previous research has revealed that HCC patients with a mixed distribution of VETC, rather than fully distributed VETC, have a worse prognosis compared to VETC-negative patients. This indicates that the prevalence of VETC as the primary mechanism of metastasis may not serve as a negative prognostic indicator but rather as an indicative marker for potential therapeutic benefit^[^45^]^. Therefore, HCC with dilated vasculature positive in arterial phase, as evaluated by CECT, may be associated with upregulated angiogenesis and VETC expression, leading to favorable treatment response to ICIs-based treatment. Overall, our findings provide novel evidence supporting dilated vasculature in arterial phase as an independent predictor of ICIs-based treatment response.

Radiomics models are often considered as opaque models, and their biological significance remains to be explored. Our GSEA of immune-related gene set suggests that radiomics model score is associated with MHC I, angiogenesis, and CD8 T-cell-related gene sets. In addition, TCIA samples classified by the model as DCB group exhibit higher p53 mutation rates, mutation burden, and neoantigen burden. The enriched biological mechanisms in the predicted DCB group have been previously demonstrated as predictive factors for the efficacy of ICIs-based treatment^[^46^]^. In summary, our model’s predictions are further validated by their congruence with established biological mechanisms, enhancing their reliability even when applied to external data that may differ somewhat from the training data.

Our study has some limitations. Firstly, it was a single-center retrospective study with a small sample size. Although we analyzed our model using TCIA samples, the lack of an external test set to evaluate the predictive ability is noteworthy. Moreover, despite employing specific strategies, the generalization ability of our models constructed with limited samples may be constrained. Therefore, prospective studies are necessary for validation purposes. As ICIs-based conversion therapy gains wide application, we will shortly carry out prospective validation at our center and in collaboration with multiple medical centers. Secondly, our model was trained using Chinese HCC patients predominantly infected with hepatitis B virus and exhibiting large tumor burdens; thus, its applicability to other countries and regions may be limited. Lastly, while bioinformatics analysis has explored the biological significance of the model, experimental verification is required to validate its biological implications.

## Conclusion

We developed a comprehensive model integrating deep learning algorithms and clinical data to accurately predict the response of ICIs-based treatment in potentially convertible HCC patients. Notably, out study elucidated the underlying biological mechanism and demonstrated potential clinical applications for conversion therapy using our model. The radiomics model presented herein can serve as a robust tool for facilitating precise decision-making and informed patient management in conversion therapy.

## Assistance with the study

We are grateful to Zhehao Xu, the general practice medical master of Sun Yat-Sen Memorial Hospital, for editing the paper format and handling the submission.
